# Expression and distribution pattern of aggrecanases and *miR-140s* in the thickened synovia of shoulder joints in rotator cuff tears: A retrospective observational study

**DOI:** 10.1097/MD.0000000000029583

**Published:** 2022-08-12

**Authors:** Takahiro Iino, Masaya Tsujii, Toru Wakabayashi, Yoshimasa Setoguchi, Masahiro Hasegawa, Akihiro Sudo

**Affiliations:** a Department of Orthopedic Surgery, Graduate School of Medicine, Mie University, Tsu, Japan; b Department of Orthopaedic Surgery, Nagai Hospital, Tsu, Japan; c Department of Orthopaedic Surgery, Toyohashi Orthopaedic Surgery Ezaki Hospital, Toyohashi, Japan; d Department of Sports Medicine, MSMC Midori Clinic, Tsu, Japan.

**Keywords:** ADAMTS, micro RNA, miR-140, osteoarthritis, rotator cuff, shoulder, synovia

## Abstract

The rotator cuff (RC) is frequently torn at the enthesis composed of fibrocartilage. We aimed to histopathologically evaluate lining layers and assess the distribution of a disintegrin and metalloproteinase with thrombospondin motifs (ADAMTS)4, ADAMTS5, and microRNA (*miR*)*-140s* in the synovia of patients with RC tears. We recruited 51 patients who underwent arthroscopic surgical treatment for full-thickness rotator cuff tears, including 26 patients with < 3 cm tear size (group N) and 25 patients with ≥ 3 cm tear size (group W). Biopsied synovia were analyzed using histological and immunohistological techniques for the presence ADAMTS4 and ADAMTS5. The layers of the synovial lining were morphologically classified into 3 grades according to the synovitis score and staining levels of ADAMTSs. The glenohumeral synovia from 8 patients with recurrent shoulder dislocation (group C) were used as controls. Furthermore, in situ hybridization was performed to evaluate the presence of *miR-140s* in patients with massive tears and recurrent shoulder dislocation. The staining levels were evaluated and analyzed based on comparison between patient groups and correlation between ADAMTS5 and *miR-140s*. Histological analysis revealed significant differences between groups W and C. ADAMTS5 and ADAMTS4 were strongly expressed in the synovial lining of patients in group W, and this expression was significantly higher than that in groups C and N. In addition, expression of ADAMTS5 was inversely correlated with that of *miR-140-3p*. This study showed that synovia from group W had a significantly higher rate of severely thickened areas with strong expression of both aggrecanases. Furthermore, the area with weak expression of *miR-140-3p* showed strong ADAMTS5 expression.

## 1. Introduction

Rotator cuff (RC) tears is the most common cause of shoulder pain and disability. The pathophysiology of RC tears has been proposed to be influenced by extrinsic factors, such as impingement wear to the acromion of the scapula, as well as intrinsic processes.^[1–3]^ Synovial tissues play an important role as a source of intrinsic factors in RC degeneration.^[4]^ Previous studies have shown that the expression of various chemical mediators is upregulated in snovial tissues in RC tears.^[5,6]^ Especially, MMP-1 was found to likely participate in the pathophysiology of RC tears because the normal tendon consists mainly of collagen fibrils.^[7,8]^

However, the RC tendon is mostly torn at the enthesis, which is composed of fibrocartilaginous tissues rich in aggrecan and type II collagen. Disintegrin and metalloproteinase with thrombospondin motifs (ADAMTS)4 and ADAMTS5 are the most efficient aggrecanases capable of degrading aggrecan.^[9–12]^ Nonetheless, no study has focused on the roles of ADAMTS4 and ADAMTS5 in the synovial tissues of patients with RC tears. Inflammation can induce catabolic activities during cartilage degradation.^[13,14]^ Furthermore, previous studies have shown that microRNA (miRNA)-140 (*miR-140*) alone regulates ADAMTS5 expression during cartilaginous degeneration,^[15–17]^ although 1 messenger RNA is regulated by multiple miRNAs.

This study aimed to histologically and immunohistochemically evaluate ADAMTSs and related factors found in the synovia in patients undergoing arthroscopic repair for RC tears.

## 2. Materials and Methods

### 2.1. Study design and setting

This was a retrospective observational study. In this study, we histopathologically examined the synovia around the RC obtained from patients who underwent arthroscopic surgery for RC tears. In addition, the synovia of the glenohumeral joint (GH) in patients with recurrent shoulder dislocation was used as a control. The primary outcome of this study was to analyze the expression and distribution of ADAMTS4 and ADAMTS5 in the synovia obtained from patients with RC tears and recurrent shoulder dislocation. The secondary outcome was to assess and compare the distribution and expression of *miR-140s* in the synovia of patients with massive RC tears and those with recurrent shoulder dislocation.

### 2.2. Patients

The study group consisted of 51 patients (21 women and 30 men) who underwent arthroscopic surgery for RC tears. The mean age of the patients was 63.1 years (range, 44 to 82 years). The exclusion criteria were as follows: previous surgery affected the current surgery during the affected surgery, previous intraarticular injection therapy, and shoulder stiffness. The patients were divided into 2 groups based on the tear size of the RC. Twenty-six and twenty-five patients were divided into groups N (tear size < 3 cm) and W (tear size ≥ 3 cm), respectively. Furthermore, synovia of the GH from 8 patients (two women and 6 men) with recurrent shoulder dislocation were used as controls (group C). Patients aged < 20 years were excluded from the study. The mean patient age in group C was 32.6 years (range: 20–58 years).

### 2.3. Sampling technique

During arthroscopic surgeries, synovia was obtained from the GH of patients with RC tears and recurrent shoulder dislocation and from the subacromial bursa (SAB) of patients with only RC tears. The synovia were immediately fixed in 10 % formalin for preparing paraffin sections for histopathological analysis.

### 2.4. Histological grading of synovia

**Figure 1.**
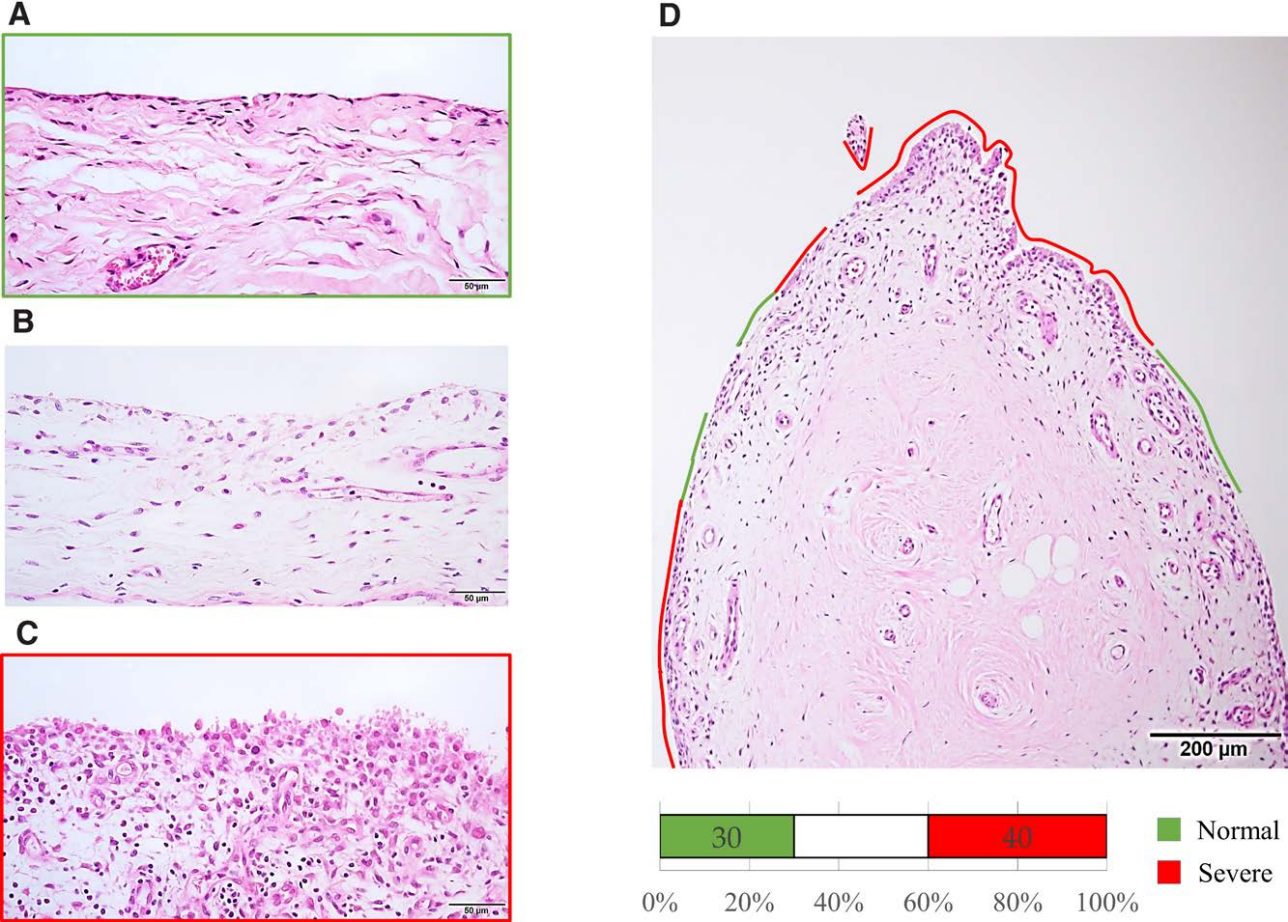
Cell layer linings of synovia in glenohumeral joints were classified into 3 types, viz. normal (A), mild (B), and severe (C), for each specimen, because the synovia showed mixed conditions of synovial thickening. In addition, lengths of the normal (green line) and severely thickened (red line) areas were measured and have been represented as the percentage of the total length (D).

The specimens were embedded in paraffin, cut into 4 µm thick sections, and stained with hematoxylin and eosin. Sections from 3 different depths for each specimen were used to obtain a representative sample of the entire specimen. The evaluation was performed by calculating the quantitative score for synovitis as established by Krenn et al.^[18]^ The cell layer linings of the synovium were classified into the following 3 types: 1 layer of lining as normal, 2 to 3 layers with lacuna between the extracellular matrix and itself as mild, and more than 4 layers with eosinophil-rich cytoplasm as severe (Fig. 1). Almost all specimens showed a cell lining layer with a mixed grade. Therefore, 2 authors (I.T. and T.M.) measured the length of the normal and severe types and the total length of the synovial lining in each specimen using image analysis software (CellSens analysis software; Olympus, Tokyo, Japan). The percentages of normal and severe types in each section were calculated by dividing the length of the normal and severe areas by the total length. The intra- and inter-observer correlation coefficients for the lengths of the 2 types were 0.88 and 0.82, respectively.

### 2.5. Sample size determination

In our hospital, the number of patients with rotator cuff tears was clearly higher than that of patients with recurrent shoulder dislocation. All samples from patients who underwent surgeries during the same period were investigated using histological analysis. To investigate the statistical powers, post hoc power analysis was performed on the results of the histological analysis based on the calculated mean and standard deviations between groups W and C using G*POWER3 (type 1 error, 0.05).^[19,20]^ Statistical powers in the areas of normal and severe thickness were 0.83 and 0.81, respectively, indicating that the number of patients included in the comparative analysis of this study was appropriate.

### 2.6. Immunohistochemical analysis

Expression levels of ADAMTS4 and ADAMTS5 were examined using antihuman ADAMTS4 rabbit polyclonal antibody (Abcam, Cambridge, MA) and antihuman ADAMTS5 rabbit polyclonal antibody (Abcam), respectively. After paraffin removal, endogenous peroxidase was inactivated using 0.3% hydrogen peroxide in methanol for 30 minutes. To determine the antigenicity of ADAMTS4 and ADAMTS5, sections were rehydrated by heating in 10 mM citrate buffer (pH 6.0) in a pressure cooker (CLIPSO 4L; Tefal, Rumilly, France) for 10 minutes. Next, the sections were kept at room temperature in the soaking solution for 30 minutes to cool; subsequently, they were incubated with antihuman ADAMTS4 and ADAMTS5 antibodies (10 µg/mL each) overnight at room temperature. Between the incubation steps, sections were dip-immersion-washed (3 × 5 min wash) in Tris-buffered saline (TBS) to eliminate excess nonbound antibodies or reagents. The antibody was diluted in 1 % bovine serum albumin/TBS to suppress nonspecific reactions. The sections were then incubated with antirabbit immunoglobulin conjugated alkaline phosphatase (ALP) using the immuno-enzyme polymer method (Histofine® Simple Stain AP; Nichirei, Tokyo, Japan). The reversible inhibitor of ALP was used 1mM levamisole solution. The reaction products were visualized using Histofine® First Red II Substrate Kit (Nichirei). The sections were counterstained with hematoxylin. For the staining levels of the synovial lining, the lengths of absent and strongly positive immunoreactivity as well as total length were measured for each specimen using the CellSens analysis software. The percentages for absent and strongly immunoreactive areas in each section were calculated by dividing the length of the absent and strongly immunoreactive areas by the total length.

### 2.7. In situ hybridization

Expression of *miR-140-5p* and *miR-140-3p* was examined by in situ hybridization staining using miRCURY LNA microRNA Detection Probe (Exiqon, Vedbæk, Denmark) in synovia of the GH from 8 patients, each with massive tears in the RC and recurrent shoulder dislocation. Double-digoxigenin (DIG) was labeled alternately (Table 1). All instruments and solutions were RNase-free. After paraffin removal, the sample was purified by treatment with 15 µg/mL proteinase K at 37 °C for 10 minutes, dehydrated with ethanol, and hybridized with 20 nM LNA probe at 55 °C for 60 minutes. After hybridization, the sections were washed with saline-sodium citrate buffer at 55 °C, and endogenous peroxidase was inactivated using 0.3 % hydrogen peroxide in methanol for 30 minutes. After washing in TBS, sections were incubated with antiDIG conjugate HRP. Then, HRP was intensified with fluorescyl-tyramide and antifluorescein conjugate HRP included in the CSA II Biotin-free Tyramide Signal Amplification System (Dako, Glostrup, Denmark). The reaction products were visualized using 0.15 mg/mL DAB solution containing hydrogen peroxide. After washing in water, the sections were counterstained with hematoxylin. The ratios of the expression area of *miR-140s* were calculated in the same manner as that used for ADAMTSs.

**Table 1:** Sequence of *has-miR-140s* and LNA probe used in microRNA in situ hybridization.

		miR Base ID	Sequence (5′-3′)
Mature miRNA	miRBase ver.22	*hsa-miR-140-5p*	*23-CAGUGGUUUUACCCUAUGGUAG-44*
		*hsa-miR-140-3p*	*62-UACCACAGGGUAGAACCACGG-82*
LNA^TM^ probe	EXIQON	*hsa-miR-140-5p*	*5DigN/CTACCATAGGGTAAAACCACT/3Dig_N*
		*hsa-miR-140-3p*	*5DigN/CCGTGGTTCTACCCTGTGGTA/3Dig_N*

### 2.8. Double staining by in situ hybridization staining and immunohistochemical staining

In situ hybridization staining was performed using the same procedure as that for *miR-140-5p* and *miR-140-3p*. The sections were incubated in 0.1M glycine hydrochloride buffer (pH2.2) for antibody removal. Immunohistochemical staining for ADAMTS5 was then performed using the same method. Finally, the sections were counterstained with hematoxylin.

### 2.9. Ethics

This retrospective observational study was approved by our institutional review board (Nagai Hospital IRB #2020022) on February 1, 2020. Written informed consent was obtained from all the patients.

### 2.10. Statistical analysis

Statistical analyses were performed using SPSS version 25 (IBM, Somers, NY) and EZR version 1.52 (Saitama Medical Center, Jichi Medical University, Saitama, Japan).^[21]^ The Kruskal-Wallis test followed by the Steel-Dwass post hoc test, were used for multiple comparisons. Correlations were analyzed using Pearson chi-squared test. *P* < .05 was considered to be statistically significant.

## 3. Results

### 3.1. Demographics of the patients

The mean age was 60.8 ± 10.2, 62.1 ± 8.6, and 32.6 ± 14.7 years for groups N, W, and C, respectively. Mean age was significantly lower in group C than in groups N and W. There was no significant difference in gender between the groups.

### 3.2. Histopathological findings of the synovial lining from the patients with RC tears and recurrent shoulder dislocation

**Figure 2.**
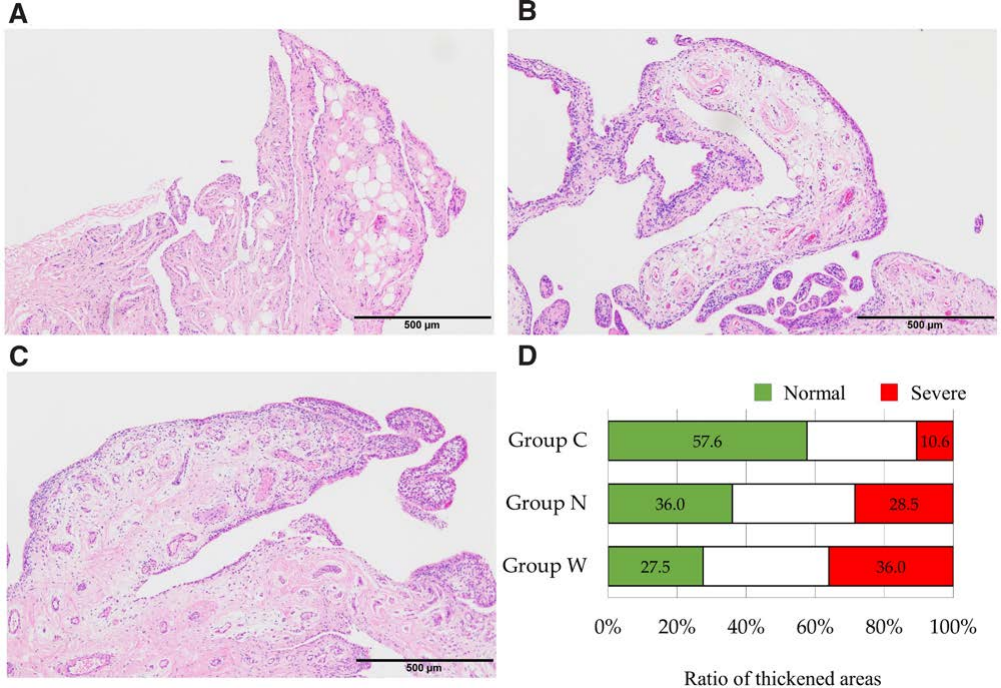
Histological findings of synovia in the glenohumeral joint from the patients with recurrent dislocation (group C; A), small tear of the rotator cuff (group N; B), and large tear of the rotator cuff (group W; C). Stacked graphs (D) show the ratio of the length of each grade to the total length of the cell layer lining of the synovia of each group.

**Figure 3.**
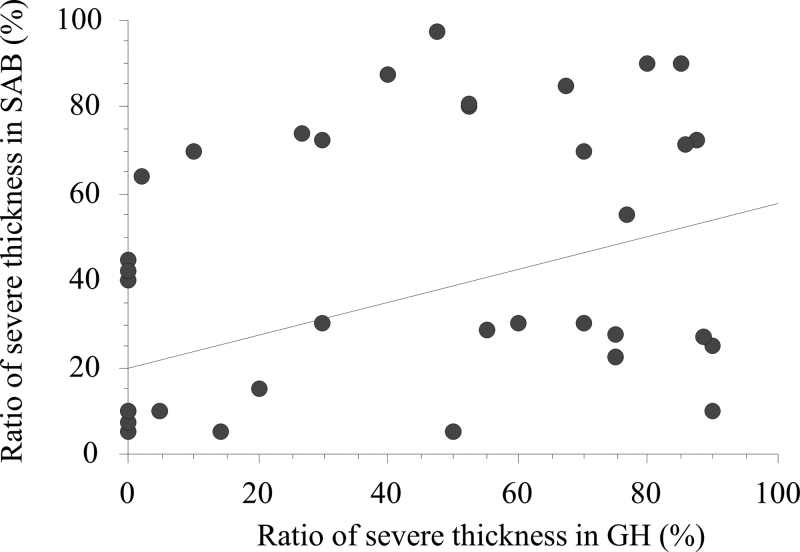
Scatterplot of liner correlation between ratios of severely thickened areas in the synovial lining between the glenohumeral joint (GH) and subacromial bursa (SAB). Pearson chi-squared test revealed a significantly positive correlation.

Histological analysis of synovia from the GH revealed that 27.5 ± 25.4 % of samples showed normal thickness of the synovial lining and 36.0 ± 31.7 % of samples showed severe thickness in group W, whereas 36.0 ± 34.1 % of samples showed normal thickness and 28.5 ± 33.8 % of samples showed severe thickness in group N. In addition, 57.6 ± 29.9 % of samples showed normal thickness and 10.6 ± 14.1 % of samples showed severe thickness in group C. The synovial lining in group W showed a significantly higher ratio of samples with severe thickness and a significantly lower ratio of samples with normal thickness than those in group C (*P* = .013 and *P* = .030, respectively; Fig. 2). Similarly, group W tended to show a higher ratio of severe thickness of the synovial lining and a lower ratio of normal thickness than those observed for group N. Moreover, there was a significant correlation between the ratio of length of the severe thickness of the synovial lining between SAB and GH (*rho* = 0.406, *P* = .004; Fig. 3), while the ratio of the normal areas was not related to the synovia between the 2 cavities.

**Figure 4.**
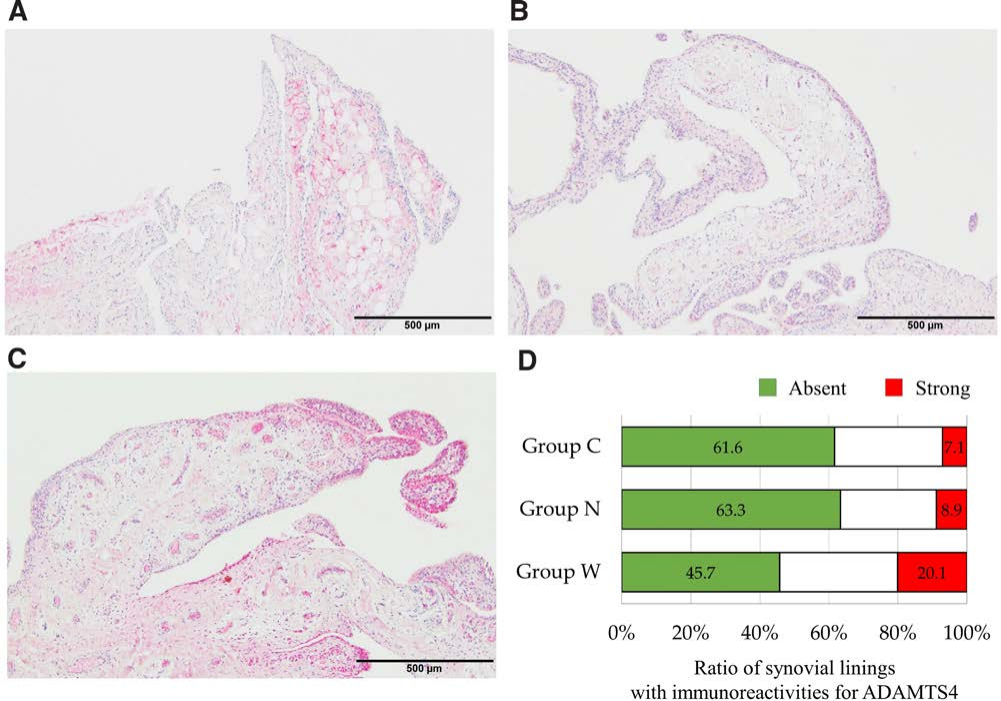
Immunolabeling for ADAMTS4 on synovia biopsied from the glenohumeral joint (GH). (A) Recurrent shoulder dislocation (group C). (B) Small tears of the rotator cuff (group N). (C) Large tears of the rotator cuff. (D) Stacked graph shows the ratio of the lengths of synovial linings with immunoreactivities for ADAMTS5 in each group.

**Figure 5.**
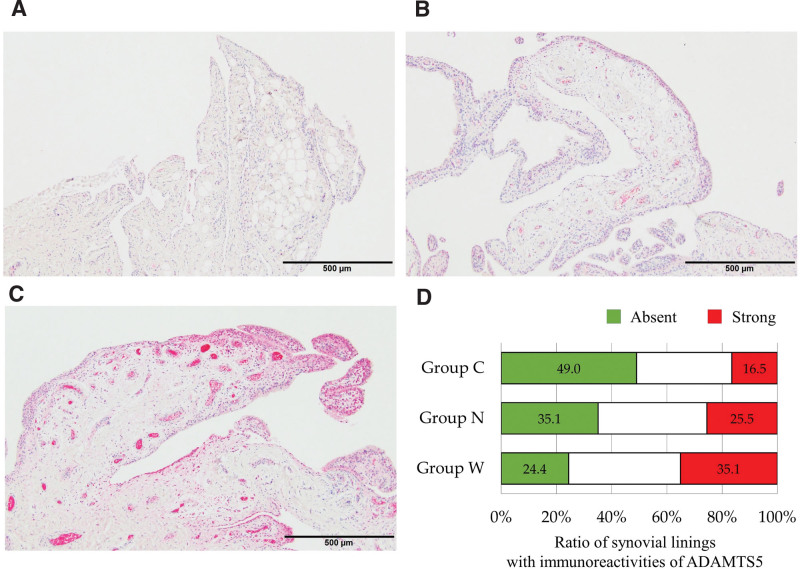
Immunolabeling for ADAMTS5 in the synovia of the shoulder joint. (A) Recurrent shoulder dislocation (group C). (B) Small tears of the rotator cuff (group N). (C) Large tears of the rotator cuff. (D) The stacked graphs show the ratio of the lengths of synovial linings with immunoreactivities for ADAMTS5 in each group.

**Figure 6.**
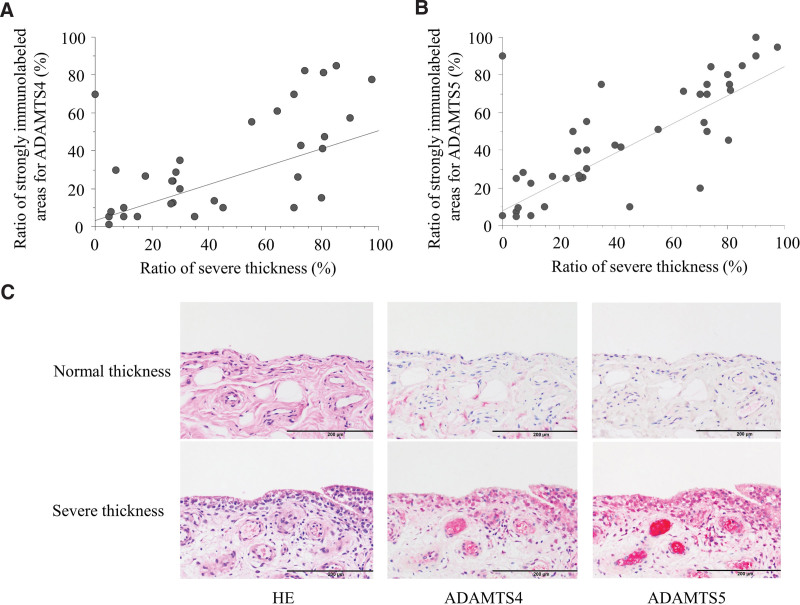
Ratios of strongly immunolabeled areas for ADAMTS4 (A) and ADAMTS5 (B) were significantly correlated with the ratio of severely thickened areas in the lining of synovia biopsied from the glenohumeral joint. (C) Areas of normal and severe thicknesses showed absent and strong immunoreactivity, respectively, for both ADAMTS4 and ADAMTS5.

In group W, the ratios of the length of strongly immunolabeled areas for ADAMTS4 (20.1 ± 19.2 %) and ADAMTS5 (35.1 ± 29.8 %) were significantly higher than those in group C (ADAMTS4, 7.1 ± 11.4 % and ADAMTS5, 16.5 ± 26.3 %) and group N (ADAMTS4, 8.9 ± 19.1 % and ADAMTS5, 25.5 ± 32.1 %; Figs. 4, 5). In addition, the ratios of the length of absent immunoreactive areas for ADAMTS4 and ADAMTS5 were significantly lower in group W than in the other groups. Furthermore, areas strongly immunolabeled for ADAMTS4 and ADAMTS5 were more likely to be found in severely thickened areas. Statistical analysis revealed a high correlation between the ratio of the lengths of areas with severe thickness and the areas strongly expressing ADAMTS4 (*rho* = 0.575, *P* < .001) and ADAMTS5 (*rho* = 0.772, *P* < .001) in the synovial lining (Fig. 6).

### 3.3. Expression and distribution of *miR-140-3p* and *miR-140-5p* in the synovia from patients with massive RC tears and recurrent shoulder dislocation

**Figure 7.**
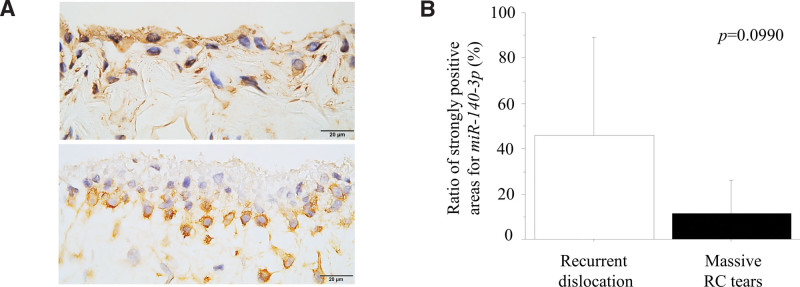
(A) In situ hybridization showed strong *miR-140-3p* expression in synovia biopsied from the shoulder joint in patients with recurrent shoulder dislocation (upper). In contrast, *miR-140-3p* expression was low in massive tears of the rotator cuff (lower). (B) Morphometric analysis showed a tendency of statistical difference in ratios of areas with strong expression of *miR-140-3p* between the synovia of patients with recurrent shoulder dislocation and those with massive tears in the rotator cuff.

**Figure 8.**
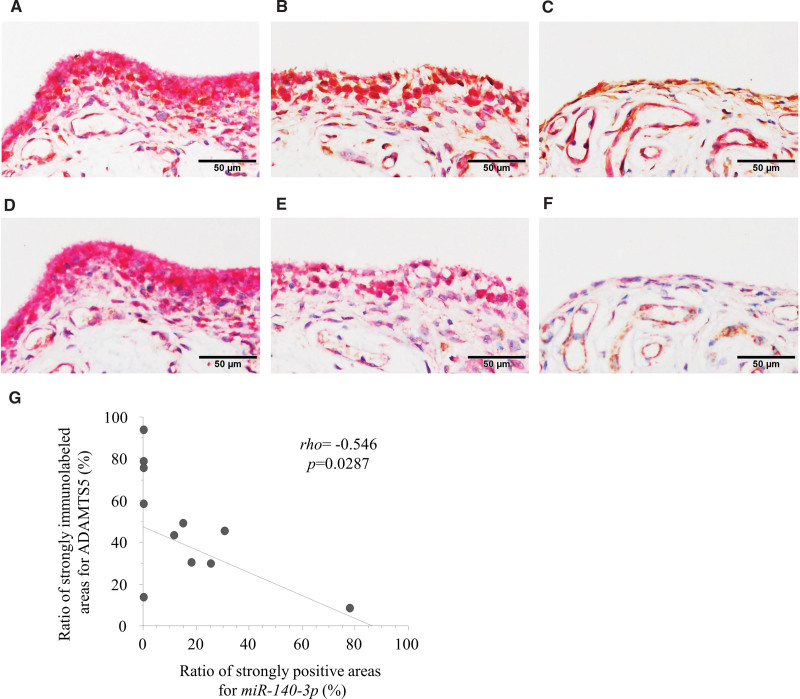
Double staining by in situ hybridization for *miR-140 (brown) -3p* (A-C) *or -5p* (D-F) and immunohistochemical staining for ADAMTS5 (red) in severely thickened areas (A, C), mildly thickened areas (B, D), and normal area (C, F) of the synovial linings. ADAMTS5 was strongly immunolabeled in the thickened linings of the synoiva, while *miR-140-3p* could not be observed in the same areas. By contrast, expression of the *miR-140-3p* were clearly shown in the normal cell layer lining of the synovia. Furthermore, immunolabeling for *miR-140-5p* were not found in the cell layer linings of the synovia but observed in the deep layers, especially on the vessels. (G) Ratio of areas with strong positivity of *miR-140-3p* was inversely correlated with the ratio of area with strong positivity of ADAMTS5.

Areas with strong expression of *miR-140-3p* were found in the cytoplasm of the synovial lining in all but 1 specimen obtained from patients with recurrent shoulder dislocation. In contrast, there were no areas with strong expression of *miR-140-3p* in the synovia of 4 of the 8 patients with massive RC injuries (Fig. 7A). Morphometric analysis showed that the ratio of strongly immunolabeled areas for *miR-140-3p* tended to be higher (*P* = .0990) in the synovial lining of the synovia from recurrent dislocation than in massive RC tears (Fig. 7B). There was a significant inverse correlation between the ratios of strong immunoreactivities with *miR-140-3p* and ADAMTS5 in the synovial linings, as shown by double staining by in situ hybridization and immunohistochemical staining methods (Fig. 8A–C, G). Furthermore, *miR-140-5p* expression was observed in the cytoplasm of fibroblasts in the deep layers, but was hardly observed in the lining of the synovia of patients with both recurrent shoulder dislocation and massive RC injury (Fig. 8D–F).

## 4. Discussion

The present study, using histological analysis, demonstrated that the synovia of the GH had significantly more areas of severe thickening of the synovial lining in patients with large tears in the RC than in those with recurrent shoulder dislocations. Furthermore, immunohistochemical analysis showed that the expression of ADAMTS4 and ADAMTS5 was distributed in the outer layer of synovial tissues. The areas with ADMAMTSs expression were significantly related to severely thickened areas. This study is the first to show the expression and distribution of *miR-140s* using in situ hybridization in the synovia of patients with massive RC tears and recurrent shoulder dislocations. A significant negative correlation was observed between the ratios of strong immunoreactivities with *miR-140-3p* and ADAMTS5 in the synovial lining.

Abrams et al have reported synovial inflammation in the GH of patients with RC injury using the same synovitis score used in this study.^[22]^ Synovial inflammation was found to be significantly greater in patients with full-thickness RC tears than in those without RC injury.^[22]^ It was not easy to evaluate the histological grade of the synovial lining because there were mixed grades and ambiguities among the grades of synovial thickness in most specimens. To overcome these problems, we developed a new evaluation technique in which the ratios of the area of only the normal thickness and severe thickness were calculated as the relative lengths divided by the total length of each specimen. As a result, reliable intra- and inter-observer correlation coefficients were recorded, suggesting that this method might represent the histological evaluation of lining thickness more accurately than the traditional method, which classifies the lining thickness into only 3 stages for each specimen. Furthermore, sampling variation, including the area for biopsy and surgery, was another limitation. Nonetheless, there was a significant correlation between the ratio of severely thickened areas of the synovia from the GH and SAB. This result may indicate that there was no significant difference in the pathological condition of the synovia, depending on the site of biopsy.

In addition, histological analysis indicated that synovial thickening in the GH was more severe in patients with large RC tears than in those with small RC tears. This finding led us to hypothesize that enlargement of the RC injury could exert a harmful influence on the integrity of the articular constitution when exposed to humoral factors from the thickened synovium. In fact, previous studies have shown the presence of catabolic factors in the cartilage and ligamentous tissues, such as matrix metalloproteinases and proinflammatory cytokines, in the synovial fluid within the GH in patients with RC injuries.^[5,23,24]^ Immunohistochemical analysis in this study also showed that the expression of ADAMTS4 and ADAMTS5 was distributed in the outer layer of synovial tissues, especially in severely thickened areas. This high expression was frequently observed in patients with large tears in the RC. As the RC is interposed between the GH and SAB, the full-thickness tear of the RC allows the humoral factors in these 2 cavities to communicate with each other. These findings suggest that the cartilage tissues of the shoulder joint could be exposed to more aggrecanases (ADAMTS4 and ADAMTS5) with the enlargement of the injured area of the RC.

The thickness of synovial tissues in the GH appears to be involved in the expression of various inflammatory mediators following RC injuries.^[5,22–24]^ The present study showed upregulation of ADAMTS4 and ADAMTS5 expression in thickened synovial tissue around the RC. Our results suggest that ADAMTS4 and ADAMTS5 are likely to be induced via an inflammatory cascade in the synovial tissues of shoulder joints following RC injuries. Furthermore, a previous study has shown that ADAMTS5 expression was tightly regulated by *miR-140* at the posttranscriptional level as well as by inflammatory mediators.^[25]^ In general, 1 messenger RNA, including ADAMTS4 and ADAMTS5, is regulated by multiple miRNAs. Therefore, changes in the expression of a single miRNA is expected to not significantly influence thought to be not important in the pathological conditions. Nonetheless, *miR-140* alone influences the pathogenesis of OA and regulates ADAMTS5 expression.^[15]^ In fact, *miR-140*^-/-^ mice show induction of early onset of spontaneous OA-like changes in their articular cartilage.^[26]^

The *miR-140* gene encodes 2 mature miRNAs, *miR-140-3p* and *miR-140-5p*, derived from the 3’and 5’strand of primary *miR-140*, respectively.^[27]^ Currently, the contribution of the lack of *miR-140-3p* and *miR-140-5p* to the phenotype remains undetermined in the cartilaginous pathophysiology. We observed that *miR-140-3p* were rarely localized in the outer layers of the synovial lining with severe grade, but clearly observed in those with normal grade. Besides, expression of the *miR-140-3p* was inversely correlated with expression of the ADAMTS5. By contrast, expression of *miR-140-5p* was hardly observed in the synovial lining, although their expression was observed in the fibroblasts of the deep layers. These findings indicate that following RC injuries, the cartilage around the RC, including the glenohumeral joint and attachment of the RC, could be degraded through the breakdown of homeostasis between ADAMTS5 and *miR-140-3p*. Therefore, *miR-140-3p* are potential therapeutic candidates for the treatment of cartilaginous degeneration in the shoulder joint in relation to RC injury. Previous studies have shown that transfection of chondrocytes and malignant tumor cells with *ds-miR-140* downregulated ADAMTS5, thus suppressing cartilage degradation, and progression and metastasis of malignant tumor cells.^[15,28]^ Transfection of the *miR-140-3p* was also reported to attenuate the joint injury in the rat model of rheumatoid arthritis.^[29]^

A limitation of the present study is that a small number of patients with recurrent shoulder dislocations were used as controls in the analysis. In addition, other analyses for assessing proteins and miRNAs, including western blotting and polymerase chain reaction, were not performed.

## 5. Conclusions

ADAMTS 4 and ADAMTS5 showed significantly higher expression in the synovia of patients with large tears of the RC than in those with recurrent shoulder dislocations. In addition, ADAMTS5 expression was found to be inversely related to *miR-140-3p* expression in the synovia in cases of RC injury.

## Acknowledgments

We thank Katsura Chiba and Kosuke Abe for their help with experimental techniques and Editage (www.editage.com) for English language editing. This study did not receive any financial support.

## Author contributions

Conceptualization: Masaya Tsujii

Data curation: Takahiro Iino, Toru Wakabashi

Investigation: Takahiro Iino, Masahiro Hasegawa, Masayoshi Setoguchi

Supervision: Masahiro Hasegawa, Akihiro Sudo

Writing - original draft: Takahiro Iino, Masaya Tsujii

Writing - review & editing: Masaya Tsujii.
